# Synchrony Vision: An RGB-D Sensor-Based System for Real-Time Monitoring and Event-Level Analysis of Interpersonal Motion Synchrony

**DOI:** 10.3390/s26144445

**Published:** 2026-07-13

**Authors:** Jinhwan Kwon

**Affiliations:** Department of Education, Kyoto University of Education, Kyoto 612-8522, Japan; kwon@kyokyo-u.ac.jp

**Keywords:** interpersonal synchrony, human motion analysis, RGB-D sensing, markerless skeletal tracking, phase difference, real-time monitoring, Kinect

## Abstract

**Highlights:**

**What are the main findings?**

- An RGB-D sensing pipeline quantified event-level interpersonal motion synchrony from markerless skeletal tracking during free dialog.
- Observed synchrony exceeded circular-surrogate baselines and remained robust across reasonable parameter settings.

**What are the implications of the main findings?**

- The system extends human motion analysis from individual movement capture to dyadic coordination monitoring.
- Exportable acceleration, timestamp, and synchrony artifacts support reproducible analysis in education, clinical, and human–robot interaction settings.

**Abstract:**

Interpersonal synchrony is a time-dependent coordination pattern in which interacting partners’ body movements become temporally aligned. This study frames interpersonal synchrony as a human motion analysis problem and presents Synchrony Vision, an RGB-D sensor-based system for real-time monitoring and event-level analysis of interpersonal motion synchrony in free dialog. The system transforms Kinect-derived skeletal positions into joint acceleration signals, applies sensor-specific conditioning, detects movement peaks, and estimates event-level phase differences between two participants within a ±1.0 s window. The operator-facing interface supports live RGB-D monitoring, acceleration visualization, joint selection, millisecond-scale phase-difference histograms, four synchrony metrics (Frequency, Lead–lag, Width, and Strength), and exportable acceleration, timestamp, peak-pairing, and summary artifacts. We evaluated the deployed pipeline on 25 Kinect-tracked dyads engaged in unconstrained conversation. Across 200.6 min comprising 245,835 frames, the system detected 2681 synchrony events. The observed event rate exceeded circular-surrogate baselines, and dyad rankings remained stable across reasonable parameter settings. Motion Energy Analysis-style cross-correlation on the same acceleration signals also confirmed above-chance synchrony but produced different dyad rankings. These findings show that RGB-D skeletal sensing can extend human motion analysis from individual movement capture to transparent, event-level quantification of interpersonal coordination.

## 1. Introduction

Sensor-based human motion analysis is increasingly central to the quantitative study of human behavior, enabling objective assessment of movement patterns in research, clinical, educational, rehabilitation, and interactive settings [1,2,3]. While traditional human motion analysis has predominantly focused on individual kinematics—such as gait, posture, or isolated upper-limb movements—the emergence of robust multi-person sensing technologies enables a shift toward analyzing social interactions. A persistent theme in this literature is interpersonal synchrony—the temporal alignment of partners’ body movements—which can be viewed as a dyadic extension of human motion analysis and has been associated with rapport, cooperation, and therapeutic processes [4,5]. To support such applications, sensing systems must balance measurement accuracy, latency, intrusiveness, scalability, and interpretability for end users [6].

Human communication combines verbal content with rich nonverbal cues, and body movement is a key channel for expressing affect, intent, and social affiliation [4,7]. A prominent nonverbal phenomenon is interpersonal synchrony, in which interaction partners’ movements become temporally coordinated during conversation and joint activity [8]. Prior work in psychology, communication, and HCI links higher movement synchrony to stronger rapport, cooperation, and more positive interaction outcomes, and it has also been discussed as a potentially useful signal in therapeutic and educational contexts where monitoring and supporting social engagement are important [9,10,11,12,13,14,15]. More broadly, coordinated body motion has been associated with social bonding, trust, empathy, and perceived interaction quality, suggesting that synchrony metrics can serve as informative behavioral markers when measured reliably [10,16,17,18].

Conventional approaches exhibit clear trade-offs. Visual observation is subjective and hard to scale; video analysis can be compute-intensive and sensitive to recording conditions; marker-based optical capture demands cost and setup; and IMU-based methods, while precise, require body-worn sensors that may alter natural behavior [19,20,21,22,23,24,25]. These constraints are particularly limiting when the goal is to capture dyadic timing relations rather than only individual kinematics, where sub-second phase relations matter for interpretation and intervention [6].

Affordable, non-invasive depth cameras such as Microsoft Kinect and Orbbec Femto Bolt provide an alternative by tracking multiple people simultaneously without on-body instrumentation. They offer RGB-D sensing, skeletal tracking, and sufficient temporal resolution for many communicative movements, making them attractive for markerless synchrony monitoring; classic work established real-time 3D skeleton estimation from depth, and subsequent analyses documented Kinect’s sensing characteristics and practical impact for human tracking [26,27,28,29]. Beyond cost and ease of deployment, such devices enable continuous measurement in ordinary rooms, which broadens the ecological validity of synchrony studies [6,29,30,31].

Building on this opportunity, phase-difference analysis has emerged as a practical and interpretable method to quantify interpersonal timing. Synchrony Vision operationalizes this idea by computing joint accelerations from tracked skeletons and detecting peaks to estimate time-lagged alignment between partners within a bounded window (±1.0 s) [32]. Because camera-derived accelerations differ from accelerometer outputs, several engineering adaptations are required: adding a constant vertical-axis offset to keep the Euclidean norm sign-stable, inverting the *X*-axis for one subject in face-to-face orientation to avoid 180° phase flips, and tuning peak-detection thresholds for Kinect data. Together, these modifications align the camera pipeline with the assumptions of the original algorithm and stabilize peak-based synchrony detection.

Empirically, the algorithmic core has been validated on metronome-paced rhythmic tasks, showing an overall event-detection accuracy of approximately 89%, and has also shown a strong correlation with a wearable-accelerometer baseline for synchrony frequency during free-dialog recordings (r ≈ 0.73) [32]. However, those results focused mainly on event detection under controlled task conditions and frequency-based agreement with a wearable reference, rather than on the deployment-condition behavior of the user-facing metrics during unconstrained free conversation. What remains to be characterized is whether the full set of user-facing metrics—Frequency, Lead–lag, Width, and Strength—behaves coherently under deployment-condition free conversation, which is the regime in which the Result workspace is intended to be used. The present manuscript evaluates the deployed, user-facing analysis pipeline under the conditions in which the software is intended to be used: unconstrained dyadic conversation. Specifically, we characterize frame-time behavior, phase-difference distributions, circular-surrogate baselines, parameter robustness, and MEA-style cross-method comparison for the four exported metrics.

For practical adoption, the computational pipeline must also be exposed through an interface that lets operators verify tracking, select target joints, inspect acceleration signals, and export auditable artifacts without relying on a separate offline workflow [33,34]. Accordingly, this study designs and implements the UI as a deployment-oriented component of the sensor-analysis method, focusing on interface functions that support measurement quality, interpretation, reproducibility, and practical use of synchrony monitoring.

This paper makes three contributions to sensor-based human motion analysis. First, it presents an RGB-D sensing pipeline that transforms markerless skeletal tracking data into acceleration-based motion signals and event-level phase-difference estimates between two interacting participants. Second, it implements these computations in an operator-facing monitoring system that supports live acquisition, joint selection, interpretable synchrony metrics, and exportable motion-analysis artifacts. Third, it evaluates the deployed pipeline on 25 Kinect-tracked free-dialog dyads, characterizing frame-time behavior, phase-difference distributions, surrogate-tested event density, dyad-level variability, parameter robustness, and correspondence with Motion Energy Analysis-style cross-correlation.

Our prior work [32] validated the core phase-difference algorithm under controlled, metronome-paced conditions against an accelerometer reference. In contrast, the present work characterizes all four user-facing metrics under unconstrained free dialog at the dyad level, introduces the deployment-oriented operator system as an integrated artifact, and adds within-dyad surrogate testing, parameter-robustness analysis, a noise/error-propagation analysis, and a parameter-matched cross-method comparison with Motion Energy Analysis.

## 2. Materials and Methods

**Figure 1:**
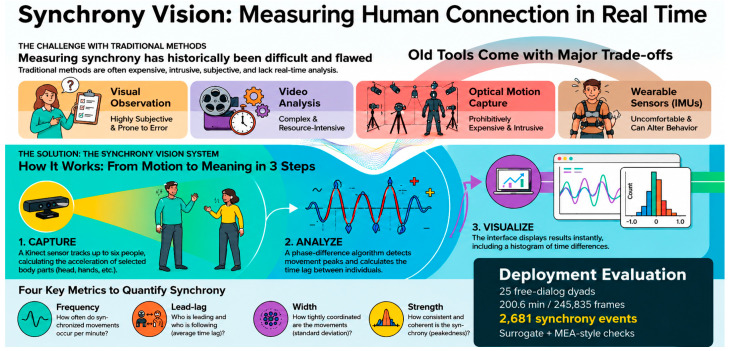
Overview of Synchrony Vision: (**top**) trade-offs of conventional synchrony measurement methods; (**middle**) Capture–Analyze–Visualize workflow from RGB-D sensing to phase-difference analytics and UI feedback; (**bottom**) four deployment-oriented synchrony metrics (Frequency, Lead–lag, Width, Strength) and deployment-evaluation context for the present study. Colors are used only to distinguish conceptual components and do not encode quantitative values.

This section describes the materials, system components, and analytical procedures used to capture, process, visualize, and evaluate interpersonal motion synchrony from RGB-D skeletal tracking. It covers the sensing hardware, acceleration derivation, phase-difference analytics, data export, user-facing workflow, and statistical comparisons with surrogate and MEA-style baselines. Figure 1 provides a high-level synopsis of Synchrony Vision as an operator-facing pipeline that converts RGB-D skeletal motion into event-level synchrony cues.

### 2.1. Hardware and Sensing

The system uses the Microsoft Kinect v2 sensor (Microsoft Corporation, Redmond, WA, USA) as a non-wearable RGB-D sensor that provides depth-based skeletal tracking in addition to color and depth streams [35]. In standard operation, the Kinect tracks up to six persons, estimating a 25-joint skeleton for each actively tracked subject over an effective range of approximately 0.5–4.5 m with a field of view of roughly 70.6° (horizontal) and 60° (vertical) [35,36,37,38]. Frames are processed at 30 fps, which is sufficient for many communicative movements such as head nods and upper-body gestures in face-to-face interaction [35,36,37,38]. By avoiding body-worn sensors, the sensing setup supports more naturalistic measurement in multi-person settings while retaining per-joint kinematic information needed for synchrony analysis.

Within the system, users can select target joints depending on the intended application. Although head and upper-body motion are commonly used for conversational synchrony, the interface supports configurable selection among the head, torso (upper body), and left/right elbows and hands, enabling the same pipeline to be applied to different body parts and interaction tasks [32]. The target joint is selected once before recording. In the standard workflow, the same joint is selected for both participants within a session, because this provides the most straightforward basis for comparing synchrony metrics across sessions. However, when the research question concerns coordination between different body parts, the system can also be configured to analyze synchrony between different selected joints across participants. Consequently, the reported metrics should be interpreted with reference to the selected joint configuration, and comparisons across sessions are most appropriate when the same joint configuration is used.

### 2.2. Data Acquisition and Kinematic Derivation

The system converts Kinect skeletal joint positions into acceleration time series through a finite-difference chain. For each tracked joint, the position sequence is sampled at the device-native frame rate and differentiated to estimate velocity and acceleration. For each frame and selected joint, the system records three-axis accelerations (*a_x_*, *a_y_*, *a_z_*) in units of $m/s^{2}$. Internally, an acceleration magnitude is standardized to reduce amplitude differences across individuals and sessions, smoothed to suppress residual depth-sensor jitter, and then used for peak detection and phase-difference estimation as defined in Section 2.5.

### 2.3. Phase-Difference Analytics and Kinect-Specific Adaptations

Synchrony is estimated using a peak-based phase-difference approach [8]. Throughout, “synchrony” denotes temporal synchrony, the temporal alignment of discrete movement events between partners; the method does not quantify the similarity of movement direction, trajectory, or kinematic pattern. Candidate motion events are defined as local maxima in the standardized acceleration magnitude. For each peak detected in one participant’s signal, the system searches for the temporally closest peak in the partner’s signal within a bounded time window (default ± 1.0 s), and the minimum peak-to-peak time difference is recorded as the phase difference. The resulting set of phase differences is summarized as a histogram and used to derive four deployment-oriented statistics: Frequency, defined as the rate of paired movement events per minute; Lead–lag, defined as the mean signed phase difference; Width, defined as the dispersion of the phase-difference distribution; and Strength, defined as a peakedness/coherence indicator derived from distribution shape [8,39,40]. In practice, the histogram provides an interpretable distributional context so that users can visually confirm whether the lead–lag tendency is concentrated or widely dispersed.

Because Kinect-derived kinematics differ from high-frequency accelerometer streams, the implementation includes three lightweight but important adaptations to align camera-based accelerations with the assumptions of the phase-difference method [32]. First, the system applies a vertical-axis offset (a numerical conditioning step, not physical gravity compensation, since the Kinect does not measure inertial acceleration) by adding a positive constant of approximately 9.8 $m/s^{2}$ to the Y component before forming norms; this prevents negative valleys from becoming artificial peaks after squaring in magnitude computation and stabilizes peak structure. Second, in face-to-face interaction, one participant’s coordinate frame can be effectively mirrored, producing a 180° phase flip in horizontal motion; the system therefore applies a face-to-face orientation correction by inverting the *X*-axis sign for one participant to restore consistent phase alignment. Third, the system uses threshold tuning for peak detection appropriate to 30-fps camera signals, setting the Kinect threshold to 0.2 (in contrast to higher thresholds used for accelerometer signals). Threshold-sweep results demonstrate that this setting detects intended nod events across multiple amplitude conditions. Together, these adaptations allow the method to preserve its original lead–lag semantics while remaining stable under depth-camera sampling and noise characteristics.

### 2.4. Data Export and Configuration

The system is organized around a transparent input–output scheme designed to support auditability and downstream analysis. On the input side, the system produces or consumes per-subject acceleration CSV files (e.g., Subject1.csv–Subject6.csv or role-based files such as Teacher.csv and Student.csv), where each row records (*a_x_*, *a_y_*, *a_z_*). Processing parameters are centralized in an external configuration file (e.g., ExtractPeak.ini), which specifies key values such as the sampling rate (FPS), peak threshold (THRESHOLD), smoothing span (SMA), phase window size (WSIZE), and histogram binning/range parameters. The documentation provides explicit schemas and examples for both the INI settings and CSV formats, enabling replication across machines and studies.

On the output side, the system exports the final analysis artifacts in human-readable CSV form. Participant-level acceleration data are saved as Subject1.csv, Subject2.csv, and additional subject-specific CSV files as needed; the phase-difference series are saved as Histogram.csv; and frame-level timestamps are saved as Time.csv.

### 2.5. Signal Processing and Phase-Difference Algorithm

This section details the end-to-end procedure that transforms per-frame joint positions into phase differences between two participants’ movements, and derives summary synchrony metrics [8,39,40].

#### 2.5.1. From Joint Positions to Standardized Acceleration

**Position → velocity → acceleration.** Let $p_{k}(t_{i})\in R^{3}$ denote the 3D position of joint k at discrete time $t_{i}$ for a participant, sampled at rate $f_{s}$ (Kinect: $f_{s}=30$ fps). The sampling interval is $\Delta t=1/f_{s}$. Velocity and acceleration are estimated by finite differences:

$$v_{k}\left(t_{i}\right)=\frac{p_{k}\left(t_{i}\right)-p_{k}\left(t_{i-1}\right)}{\Delta t}$$

$$a_{k}(t_{i})=\frac{v_{k}(t_{i})-v_{k}(t_{i-1})}{\Delta t}$$

**Norm and z-score normalization.** To obtain a scalar signal suitable for peak-based event extraction, we compute the acceleration magnitude:

$$m_{k}(t_{i})={\left\|a_{k}(t_{i})\right\|}_{2}=\sqrt{a_{x}(t_{i}{)}^{2}+a_{y}(t_{i}{)}^{2}+a_{z}(t_{i}{)}^{2}}$$

Because motion amplitude varies across participants, joints, and sessions, the magnitude is standardized using z-score normalization over the analysis interval T:

$$\mu_{m}=\frac{1}{\left|T\right|}\sum_{t_{i}\in T}m_{k}\left(t_{i}\right)$$

$$\sigma_{m}=\sqrt{\frac{1}{|T|-1}\sum_{t_{i}\in T}{\left(m_{k}(t_{i})-\mu_{m}\right)}^{2}}$$

$$m_{k}^{*}(t_{i})=\frac{m_{k}(t_{i})-\mu_{m}}{\sigma_{m}}$$

Given the inherent spatial jitter and depth-estimation noise characteristic of consumer-grade depth sensors, the standardized signal is smoothed using a short moving average. This sensor-specific conditioning is crucial to prevent high-frequency artifacts from being falsely detected as motion peaks, while preserving salient movement bursts:

$${\tilde{m}}_{k}(t_{i})=\frac{1}{W}\sum_{r=0}^{W-1}m_{k}^{*}(t_{i-r})$$

where W is the SMA window length (default W=11). For Kinect at 30 fps, W=11 corresponds to approximately 0.37 s, and may be tuned depending on motion speed and noise conditions.

#### 2.5.2. Peak Detection as Motion Events

Motion events are defined as local maxima of the smoothed standardized signal. A sample $t_{i}$ is considered a peak if:

$${\tilde{m}}_{k}(t_{i-1})<{\tilde{m}}_{k}(t_{i})\geq {\tilde{m}}_{k}(t_{i+1})\;\mathrm{and}\;{\tilde{m}}_{k}(t_{i})\geq \theta$$

where θ is an amplitude threshold. In practice, θ is modality-dependent: Kinect-derived accelerations at 30 fps require a lower threshold (default θ=0.2) than standardized high-rate accelerometers.

#### 2.5.3. Kinect-Specific Signal Adaptations

Skeleton-derived kinematics differ from wearable accelerometer streams, and three lightweight adaptations are used to align the camera pipeline with the assumptions of the phase-difference method.

**Vertical-axis offset for norm stabilization.** The Kinect does not measure inertial acceleration; therefore, this step should not be interpreted as physical gravity compensation. Rather, it is a numerical conditioning offset. Before computing the Euclidean acceleration magnitude, the system adds a positive constant to the vertical component (*Y* axis). This keeps the vertical component on a positive baseline and reduces the risk that negative vertical excursions are rectified by the Euclidean norm and detected as spurious peaks after squaring. The specific magnitude of the constant is not critical, provided that it exceeds typical vertical-acceleration excursions; we use a value on the order of 9.8 for historical continuity with the original implementation.

$$a_{y^{\prime }}(t_{i})=a_{y}(t_{i})+g,\;g\approx 9.8\;{m/s}^{2}$$

The acceleration magnitude is then computed using $a_{y^{\prime }}(t_{i})$ in place of $a_{y}(t_{i})$.

**Face-to-face orientation correction.** In face-to-face interaction, one participant’s horizontal axis may be mirrored by the coordinate convention, leading to an apparent 180° phase flip. The system therefore inverts the sign of the X component for one participant prior to magnitude computation, restoring consistent phase alignment for horizontal motion.

**Threshold tuning for 30-fps camera signals.** Because Kinect signals exhibit lower apparent amplitude after differentiation and smoothing, θ is tuned to a lower range (default 0.2). Practical tuning is performed by verifying that intended event counts (e.g., nods in rhythmic tasks) are recovered robustly across amplitude conditions.

These adaptations preserve the original lead–lag semantics of the algorithm while improving stability under depth-camera sampling and noise characteristics.

#### 2.5.4. Phase-Difference Pairing and Deduplication

Let $P_{A}=\{t_{i}^{A}\}$ and $P_{B}=\{t_{j}^{B}\}$ denote the detected peak times for participants A and B, respectively. We define a maximum allowable lag $\tau_{max}$ (default 1.0 s). For each peak $t_{i}^{A}$, we search for the closest counterpart in $P_{B}$ within the window:

$$t_{j}^{B}=arg\underset{t\in P_{B}}{min}|t-t_{i}^{A}|\;s.t.\;|t-t_{i}^{A}|\leq \tau_{max}$$

and record the signed phase difference:

$$\Delta \phi_{ij}=t_{j}^{B}-t_{i}^{A}$$

where $\Delta \phi_{ij}<0$ indicates that B leads A, whereas $\Delta \phi_{ij}>0$ indicates that A leads B. Because multiple peaks may fall within $\tau_{max}$, the implementation performs pairing in both directions (A-anchored and B-anchored) and then removes duplicates to enforce one-to-one matching and prevent double counting.

**Edge cases.** If no counterpart peak exists within $\tau_{max}$, the anchor peak is discarded. If multiple candidates tie in temporal distance, the closest in time is chosen; optionally, peak amplitude can be used as a secondary tie-breaker when stored.

#### 2.5.5. Output Metrics and Visualization

Given the set $\left\{\Delta \phi_{ij}\right\}$:

**Density (activity)** is defined as the synchronization event rate per minute:

$$\mathrm{Density}=\frac{N_{\mathrm{pairs}}}{T_{min}}$$

**Lead–lag (mean temporal offset)** is defined as the mean phase difference and captures both the magnitude and sign of the average temporal offset:

$$\bar{\Delta \phi }=\frac{1}{N}\sum_{n=1}^{N}\Delta \phi_{n}$$

A negative $\bar{\Delta \phi }$ indicates that participant B tends to lead participant A, whereas a positive value indicates that participant A tends to lead participant B.

**Width (variability)** is defined as the dispersion of the phase-difference distribution, computed as the standard deviation of the time lags $\sigma_{\Delta \phi }$.

**Strength (coherence)** is defined as the Pearson kurtosis of the phase-difference distribution, computed from the fourth central moment normalized by the squared variance. Higher values indicate a more sharply peaked distribution of Δ*ϕ* values; because it is distributional, Strength is interpreted together with Frequency and Width rather than as a standalone validation score.

Histogram of Δϕ supports visual inspection of unimodality versus spread, and the horizontal axis is represented in milliseconds in the current UI to facilitate direct interpretation as conversational time delays.

#### 2.5.6. Noise Conditioning and Error Propagation

Because acceleration is obtained by double finite differencing of position, high-frequency measurement noise is amplified. For central second differences, the analytic noise gain is √6/Δt^2^, which at 30 fps is approximately 2.2 × 10^3^ times the position-noise standard deviation. The z-score and smoothing stage are designed to control this. The moving average (W = 11 at 30 fps) has a −3 dB cutoff of 1.22 Hz, passing the conversational-motion band (<2 Hz) while attenuating the differentiation-amplified high-frequency content. Measured across the 50 signals of the corpus, smoothing removes 97.4% ± 0.3% of the power in the 2–15 Hz noise band while retaining 77.5% ± 3.1% of the 0.1–2 Hz motion-band power, improving the motion-to-noise power ratio by a factor of about 30. The residual quiescent noise floor, estimated as the 10th-percentile one-second-window standard deviation of the standardized signal, is 0.079 ± 0.056 in z units, well below the peak-detection threshold θ = 0.2; noise-driven false peaks are therefore structurally suppressed (Figure S2).

### 2.6. User Interface and Export Workflow

This section describes the user interface (UI) that operationalizes phase-difference–based synchrony analytics for end-users. The UI is organized into two primary workspaces—Measurement and Result—and is complemented by a small set of configuration and export mechanisms. The central design intent is to keep complex analytics visible and interpretable while ensuring that the operator workflow remains lightweight during time-critical data acquisition.

#### 2.6.1. Design Rationale

The UI was designed around three practical requirements. First, it keeps live camera feedback, acceleration traces, recording controls, and system messages on a stable measurement surface so that operators can verify state without context switching. Second, it exposes only frequent acquisition decisions—start/stop recording, target-joint selection, and graph scaling—while keeping advanced analysis parameters in an external configuration file. Third, it preserves auditable outputs so that every summary shown in the Result workspace can be checked or reanalyzed outside the application [33,34].

#### 2.6.2. Measurement Workspace

Operationally, the UI supports a short and repeatable workflow: Launch → Recognition → Measurement Settings → Start (REC) → Stop → Results → Export. During recognition, color-coded body overlays and Body1–Body6 status indicators help operators verify which SDK-assigned tracking index corresponds to each physical participant before recording begins. This explicit recognition step reduces the risk of swapping participant roles in exported files when SDK-assigned body indices change or appear ambiguous at first detection. Target joints are then selected and visually confirmed, after which recording starts with persistent REC feedback. When recording ends, the software automatically executes the phase-difference analysis and transitions to the Result workspace for interpretation and export. The current interface displays color-coded tracking overlays on the live camera view and plots the selected joint’s acceleration in real time. It does not render a separate articulated skeletal model or provide post hoc motion replay. These functions are planned as future interface enhancements.

**Figure 2:**
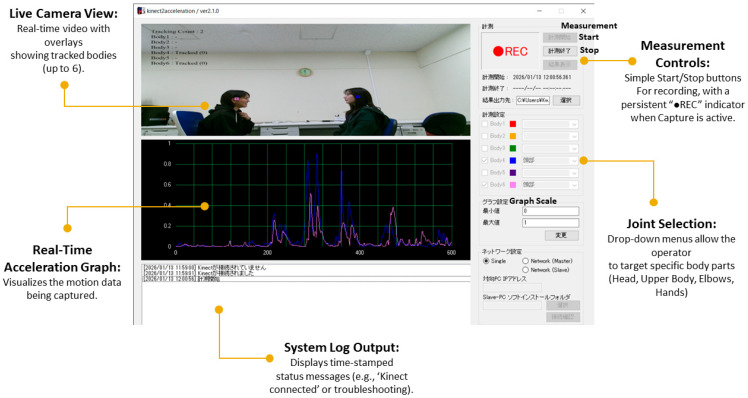
Measurement workspace of Synchrony Vision (annotated screenshot). The Measurement screen integrates a live RGB-D camera view with tracking overlays, real-time acceleration traces for selected joints, recording controls with a persistent REC indicator, per-subject joint-selection controls, graph-scale controls, and a time-stamped system log for in situ status monitoring and troubleshooting during capture.

The Measurement screen presents a single integrated surface comprising (i) the live camera view with status overlays, including Tracking Count and Body1–Body6 “Tracked/–“ flags; (ii) real-time acceleration plots; (iii) a control panel for measurement; (iv) per-subject joint selection controls and *y*-axis range settings to keep salient peaks visible; and (v) a log pane, as shown in Figure 2. A dedicated log pane streams system messages and state transitions, supporting troubleshooting without requiring users to exit the main screen.

The camera display reports the current Tracking Count and Body1–Body6 status flags, while color-coded overlays help the operator map SDK-assigned body indices to physical participants. Per-subject selectors specify the target body part, allowing the same sensing pipeline to be applied to head, torso/upper-body, elbow, or hand motion depending on the study design. The acceleration plots visualize per-subject signals in real time, and graph-scale controls let the operator keep movement peaks within view when motion amplitudes differ across participants or tasks. Because the phase-difference method depends on event peaks, this immediate visual check is part of the measurement-quality workflow rather than only a display feature. Acceleration is the real-time signal displayed in the current interface because the peak-based event detector operates on the acceleration-derived signal. Position and velocity are computed internally as intermediate quantities in the finite-difference chain, but they are not plotted live in the current version. Start/Stop/Show Results buttons manage the transition from acquisition to analysis. A persistent REC indicator and timestamped log messages mark recording state, device status, recognition events, file I/O, and analysis initiation, which helps operators detect setup or tracking problems while the session is still recoverable.

#### 2.6.3. Result Workspace and Exported Artifacts

**Figure 3:**
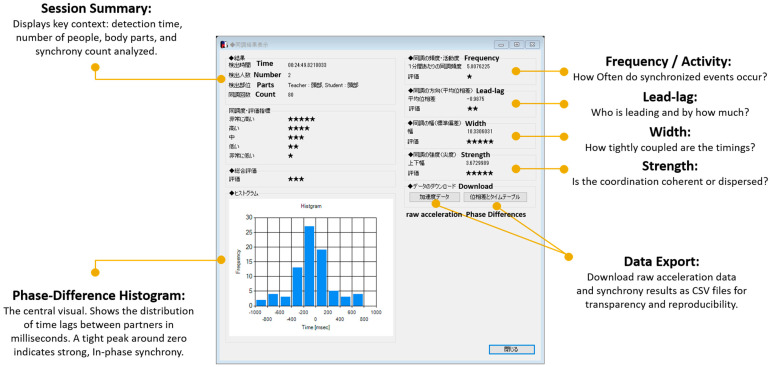
Result workspace of Synchrony Vision (annotated screenshot). After recording, the Result screen summarizes session context and presents four deployment-oriented synchrony metrics—Frequency/Activity, Lead–lag, Width, and Strength—together with a millisecond-scale phase-difference histogram and export controls for acceleration, timing, peak-pairing, and summary artifacts.

After recording ends and at least two participants are tracked, the software executes the phase-difference analysis and presents results in the Result workspace (Figure 3). The screen reports session metadata, selected body parts, synchrony count, and a millisecond-scale histogram of signed phase differences.

The real-time component of the system is the live acceleration display and peak monitoring during recording; the four summary metrics and the histogram are computed once, after recording stops, rather than streamed continuously. In typical operation on the development environment used for the present recordings, the Result workspace was generally available within approximately 5 s after pressing Stop.

Four metric cards summarize complementary properties of the event-level synchrony distribution. Frequency reports how often partner-matched movement events occur per minute. Lead–lag, defined as the mean signed phase difference, captures both the magnitude of the temporal offset and which participant tends to lead. Width, measured by the standard deviation of phase differences, reflects the temporal dispersion of alignment. Strength, derived from a kurtosis-related indicator, represents the peakedness and consistency of the phase-difference distribution. These cards support quick inspection while preserving continuous values for downstream analysis.

The histogram uses milliseconds rather than frames on the horizontal axis, making lead–lag values interpretable at conversational time scales. This display also helps users distinguish a near-zero, bidirectional distribution from a broad or systematically shifted distribution.

One-click export buttons save participant-level acceleration files (Subject1.csv, Subject2.csv), phase-difference series (Histogram.csv), and frame-level timestamps (Time.csv) as human-readable CSV files. Keeping these outputs human-readable supports replication, reanalysis with external scripts, and comparison with alternative synchrony methods.

#### 2.6.4. Configuration, Pre-Flight Checks, and Recoverability

To keep routine operation simple, algorithm parameters are centralized in an external INI file rather than exposed as on-screen controls. Key settings include FPS, the peak-detection threshold (default θ = 0.2 for Kinect signals), the SMA smoothing window (default W = 11 samples), the maximum pairing window (default $\tau_{max}$ = 1.0 s), histogram range/binning, and optional threshold values for metric ratings. This separation lets expert users standardize settings across studies while allowing non-experts to use fixed deployment defaults [33,34].

**Figure 4:**
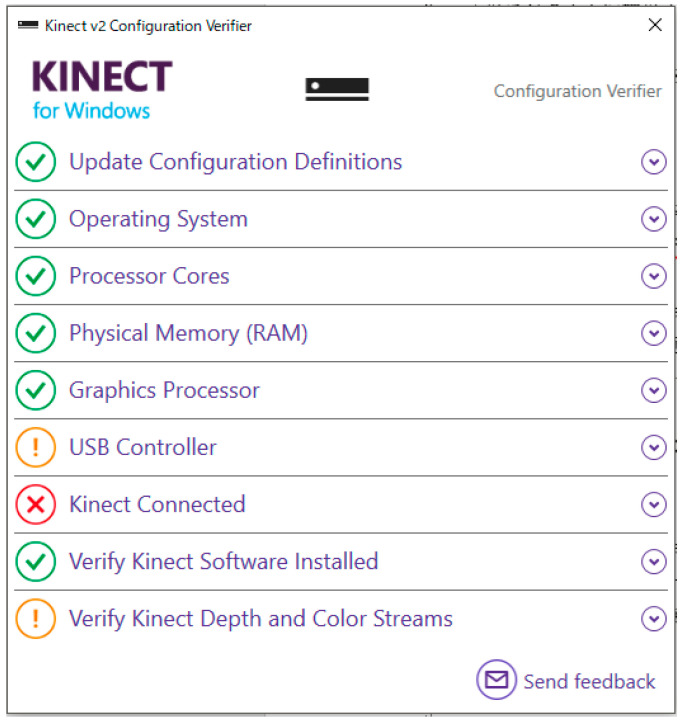
Configuration Verifier (pre-flight check screen). The verifier reports system readiness for Kinect operation by checking configuration definitions, operating system, CPU cores, physical memory, graphics processor, USB controller bandwidth, device connection status, Kinect software installation, and availability of depth/color streams, using pass/warn/fail indicators to support rapid diagnosis before running the application.

Figure 4 shows the Kinect Configuration Verifier used for pre-flight checks of hardware, drivers, USB bandwidth, and stream availability. This pre-flight step is intended to reduce installation and setup failures before field recording begins. Reporting pass/warn/fail status before acquisition reduces the risk of silent misconfiguration that would compromise motion recordings.

During operation, the system progresses through visible states—Idle, Tracking, Recording, Analysis, and Results—marked by tracking indicators, the REC badge, and timestamped log messages. Common recoverability paths, such as checking power/USB connection or re-confirming body-role mapping, are therefore available within the measurement workflow rather than requiring developer tools.

### 2.7. Evaluation Methods

Evaluation focused on deployment-condition behavior rather than re-reporting the prior controlled validation of the system implementation [32]. The practical question was whether the same timestamp-aware phase-difference computation that populates the Result workspace produces coherent Frequency, Lead–lag, Width, and Strength values when applied to long, unconstrained dyadic conversations. We therefore evaluated the pipeline on a Kinect-acquired corpus at the dyad level, examined effective frame-time characteristics, tested event density against circular-surrogate baselines, assessed parameter robustness, and compared the event-based outputs with Motion Energy Analysis-style windowed cross-correlation [41,42].

#### 2.7.1. Procedure

Twenty-five dyads of native Japanese speakers (50 participants, 25 conversational pairs) engaged in unconstrained, seated, face-to-face free dialog. Participants were instructed only to talk freely; no task, topic, or pacing was imposed. Head motion was captured with a Microsoft Kinect sensor positioned at conversational distance and operated using Synchrony Vision version 2.1.0, developed in-house, with Microsoft Kinect for Windows SDK 2.0. For each participant, the Kinect SDK reported skeletal joint positions at the device-native rate, which were converted to tri-axial acceleration time series (*a_x_*, *a_y_*, *a_z_*) via the finite-difference chain defined above and exported as CSV through the standard UI export path (Subject1.csv, Subject2.csv) along with per-frame timestamps (Time.csv). This study was approved by the Ethics Committee of Kyoto University of Education (Approval No. 1805) and conducted in accordance with the Declaration of Helsinki.

#### 2.7.2. Analysis Pipelines

**Phase-difference pipeline.** The pipeline applied to every dyad is the one described in Section 2.1, Section 2.2, Section 2.3, Section 2.4, Section 2.5 and Section 2.6; no per-dyad tuning was performed, and all parameter values match the deployment defaults. Before synchrony analysis, inter-frame intervals were computed from Time.csv for each dyad, and effective frame-rate summaries were derived from these timestamp differences. The phase-difference analysis used the original frame sequence, and all timing computations—pairing windows, phase-difference values, and Frequency denominators—were based on Time.csv (wall-clock) timestamps. The resulting phase-difference outputs were then validated in three steps: first, by comparing observed event density against a within-dyad chance baseline; second, by characterizing per-dyad variability in the four UI metrics; and third, by testing the robustness of these metrics to reasonable changes in the peak-detection threshold, smoothing window, and pairing window.

**Motion Energy Analysis pipeline.** To enable a direct, parameter-matched comparison with the canonical Motion Energy Analysis (MEA) framework [41,42], we additionally applied the MEA windowed cross-correlation conventions to the same z-scored, smoothed acceleration magnitudes that were used as the input to the deployed phase-difference pipeline. We used the exact parameter set advocated by Ramseyer and Tschacher [41] and restated as the recommended default in Ramseyer’s primer [42]: 1 min non-overlapping windows (winSec = 60 s, incSec = 60 s) and a maximum lag of ±5 s. Within each window, we computed the cross-correlation function and took the absolute value of the peak *r* within the ±5 s lag range; the per-session synchrony index was the mean of these absolute peaks across windows, which is the quantity that the MEA literature reports as the dyad-level “synchrony” value. To compare observed synchrony with chance, we generated 100 pseudosynchrony surrogates by window-wise shuffling of one participant’s time series, following the bootstrap convention introduced by Ramseyer and Tschacher [41] and described in detail by Moulder et al. [43]. For each dyad, the within-pair Cohen’s *d* against the surrogate distribution was computed in the same manner as in the MEA literature, enabling a direct numerical comparison of effect sizes with their published values.

#### 2.7.3. Supplementary Quality, Noise, and Agreement Analyses

To address timing quality, noise attenuation, and cross-method agreement, three supplementary analyses were conducted. First, frame-time quality was assessed from Time.csv by computing dyad-level median and mean effective frame rates, the distribution of inter-frame intervals, and the maximum timestamp gap for each dyad. These analyses were used to evaluate whether timestamp irregularities or long frame gaps could affect event-level pairing within the ±1.0 s window.

Second, because acceleration was derived from Kinect skeletal positions through finite differences, we examined the smoothing and noise characteristics of the acceleration-derived signals. Specifically, we evaluated the frequency response of the W = 11 simple moving average filter, estimated the proportion of high-frequency power removed in the 2–15 Hz band, and compared the quiescent noise floor with the deployment peak-detection threshold θ = 0.20.

Third, to evaluate whether the phase-difference approach and MEA-style analysis could be treated as interchangeable dyad-level measures, PDA Frequency and MEA |r| were z-standardized across dyads and compared using Pearson correlation, ICC(2,1) for absolute agreement, and Bland–Altman analysis. The corresponding supplementary results are presented in Figures S1–S3.

## 3. Results

### 3.1. Frame-Time Characteristics

The effective frame-time series was consistent with typical Kinect behavior: nominal 30 fps acquisition with irregular intervals introduced by occasional frame drops during RGB-D streaming. Across the 25 dyads, Time.csv contained 245,835 frame-level timestamps corresponding to 200.6 min of paired Kinect-tracked interaction, with a mean recording length of 8.0 ± 0.8 min per dyad. The corpus-wide arithmetic mean effective frame rate was 20.5 ± 2.7 Hz, whereas the dyad-level median frame-rate estimate was 27.8 ± 1.3 Hz. This indicates that most inter-frame intervals remained close to the nominal Kinect rate, while occasional timestamp gaps lowered the arithmetic mean. The median inter-frame interval was 36.0 ms, compared with the nominal 33.3 ms interval. Intervals exceeding 1.5 times the nominal interval accounted for 34.3% ± 8.7% of intervals, and the estimated missing-frame fraction was approximately 35% per dyad. Importantly, long timestamp gaps exceeding 500 ms occurred in only 7 of 245,810 inter-frame intervals (0.00285%), and the worst-case inter-frame gap across all 25 dyads was 0.57 s, which remained below the ±1.0 s pairing window (Figure S1).

The participant-level acceleration files were row-aligned with Time.csv for all dyads, and no NaN values were detected. Because all timing computations used the recorded wall-clock timestamps rather than an assumed uniform 30 fps frame index, frame drops reduced the effective sampling density but mitigated timing bias that would arise from assuming perfectly uniform sampling. We also note a logging limitation: the deployed software recorded per-frame timestamps but not SDK per-joint TrackingState values. Therefore, joint-confidence and occlusion flags could not be reconstructed for this corpus. Logging TrackingState and selected-joint confidence is therefore recommended for future deployments and is listed as a limitation.

### 3.2. Distribution of Phase Differences

**Figure 5:**
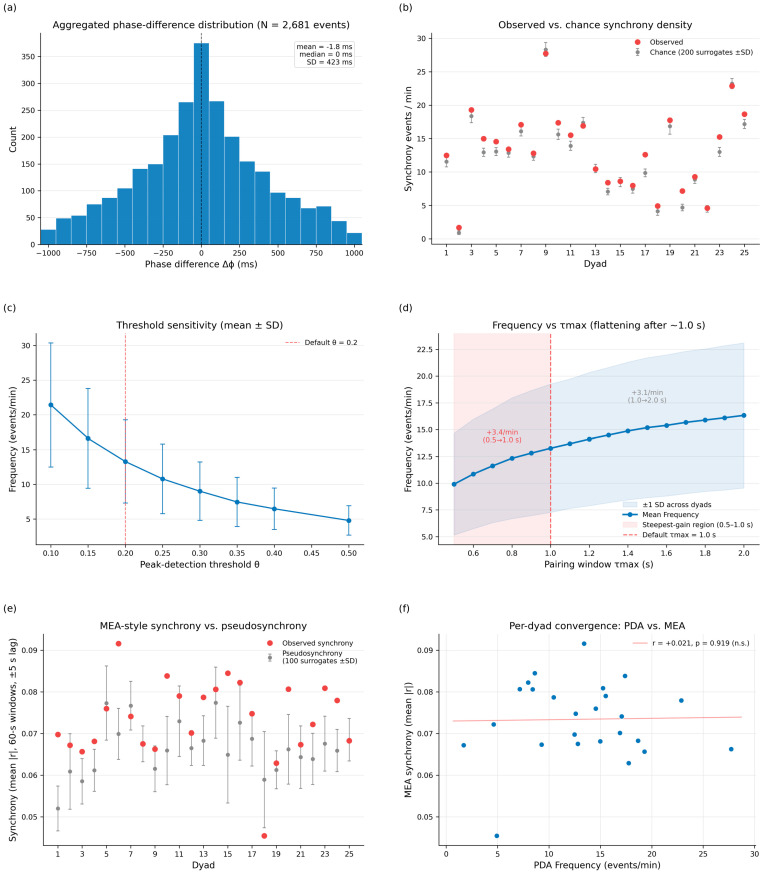
Deployment-condition evaluation of Synchrony Vision on 25 Kinect-tracked free-dialog dyads. (**a**) Aggregated distribution of signed phase differences across 2681 detected synchrony events, computed from Time.csv wall-clock timestamps. (**b**) Per-dyad observed synchrony density compared with the mean ± SD of 200 within-dyad circular-surrogate baselines. (**c**) Sensitivity of mean Frequency to the peak-detection threshold θ across [0.10, 0.50]; error bars indicate inter-dyad variability, defined as the SD of per-dyad Frequency across the 25 dyads at each threshold. (**d**) Sensitivity of Frequency to the maximum pairing window $\tau_{max}$ across [0.5, 2.0 s]. (**e**) MEA-style synchrony compared with 100 window-wise pseudosynchrony surrogates using 1 min windows and a ±5 s lag. (**f**) Relationship between phase-difference Frequency and MEA |r|, showing minimal dyad-level convergence.

Across the 25 Kinect-tracked dyads, the phase-difference pipeline identified 2681 matched synchrony events, using wall-clock timestamps from Time.csv for all temporal computations. Aggregating the signed phase differences across all events yields a unimodal distribution centered close to zero (Figure 5a; mean = −1.8 ms, median = 0 ms, SD = 423 ms). Of all detected events, 42.5% fell within ±200 ms of zero, 54.8% within ±300 ms, and 74.4% within ±500 ms, which is the time scale typically reported for conversational co-motion in prior synchrony work [8,39]. The near-zero center indicates the absence of a systematic lead–lag bias across the corpus, which is the expected pattern for unstructured dyadic conversation in which neither participant occupies a fixed leader role. The roughly symmetric tails on either side of zero, together with the millisecond axis of the histogram exposed in the Result workspace, allow operators to visually confirm that the analyzed session approximates a bidirectional mutual-coordination process rather than a unidirectional driving relation.

### 3.3. Comparison Against a Chance Baseline (Phase-Difference Pipeline)

To assess whether the observed event rate exceeds what would be expected from independently timed motion in each pair, a within-dyad surrogate test was conducted. For each dyad, participant B’s peak times were circularly shifted by a uniformly random offset within the recording window, the pairing procedure was repeated, and 200 such surrogates produced a chance distribution of event densities. Observed density exceeded the surrogate mean in 24 of 25 dyads (Figure 5b), and exceeded the upper 95% of surrogates in 11 of 25 dyads. Across the corpus, the observed Frequency (M = 13.31 events/min) was significantly higher than the chance density (M = 12.36 events/min; Wilcoxon signed-rank, *p* < 0.001; mean within-pair difference = +0.95 events/min, SD = 0.95; Cohen’s *d_z_* = 1.04). The non-trivial chance level (~12/min) reflects the fact that two people moving spontaneously at conversational pace will produce some accidental coincidences within a ±1.0 s window once the rate of detected peaks is high; the systematic excess of observed over chance therefore quantifies temporal structure above the circular-shift baseline rather than mere independent peak matching.

### 3.4. Per-Dyad Metrics and Inter-Pair Variability

At the corpus level, Frequency averaged 13.31 ± 5.98 events/min (median = 13.40, range 1.69–27.72). Dyad-level Lead–lag values were centered near zero and were not significantly different from zero, whereas median absolute lag averaged 263.42 ± 51.08 ms, Width averaged 422.63 ± 31.64 ms, and Strength averaged +2.78 ± 0.36. Three properties of the dyad-level metrics are worth noting. First, the per-dyad Frequency spans more than an order of magnitude (1.7–27.7 events/min), indicating that the metric carries clear dyad-level signal rather than collapsing to a corpus-wide constant; this is the property required for the metric to function as a comparator on the Result screen. Second, Lead–lag was small in absolute value, confirming that the deployed interface’s lead–lag rendering does not show a spurious corpus-level bias when used on neutral free conversation. Third, Width is comparatively stable across dyads (coefficient of variation 7.5% vs. 45.0% for Frequency), indicating that the temporal spread of synchrony is a relatively conserved property of free dialog. The magnitudes obtained here are consistent in order with those recently reported on independent typically developing dyads in a clinical-context study using the same algorithmic family [40]. For interpretation, Frequency represents the number of temporally aligned movement events per minute. Thus, under an identical configuration, a dyad with Frequency = 20 events/min produced approximately twice as many partner-matched movement peaks within the ±1.0 s window per minute as a dyad with Frequency = 10 events/min. Because the absolute count depends on the selected joint, threshold θ, smoothing window, and pairing window, Frequency values are configuration-relative and should be compared only when these settings are held constant. A higher Frequency value should not, by itself, be interpreted as indicating better interaction quality.

### 3.5. Robustness to Parameter Choices and Noise Attenuation

Although the deployed system fixes its analysis parameters in an external INI file (θ = 0.20, W = 11, $\tau_{max}$ = 1.0 s), we examined the sensitivity of the corpus-level results to each parameter to characterize the parameter dependence of the UI metrics. Varying θ across [0.10, 0.50] produced a monotonic decrease in mean Frequency from 21.4 to 4.7 events/min (Figure 5c), while leaving the rank order of dyads largely intact (Spearman’s ρ = 0.97 between θ = 0.20 and θ = 0.30). Varying the smoothing window W over [5,21] produced an analogous monotonic decrease in Frequency, again preserving rank order. Varying $\tau_{max}$ over [0.5, 2.0 s] increased Frequency (Figure 5d), with most of the gain concentrated between 0.5 and 1.0 s, after which the curve flattened. This pattern is consistent with the practical use of the ±1.0 s window as a deployment default for the present dataset, while also indicating that this value should not be regarded as an empirically optimized timescale. Taken together, these results indicate that the absolute magnitudes of the metrics scale predictably with their underlying parameters, whereas the relative ordering of dyads is preserved across reasonable parameter neighborhoods. This preservation of rank order supports comparison-oriented use of the Result workspace under fixed parameter settings, while also indicating that absolute metric values should be interpreted as configuration-relative.

Because the peak-detection pipeline uses acceleration-derived signals computed from Kinect skeletal positions, we also examined the effect of smoothing and the separation between the quiescent noise floor and the deployment threshold. The W = 11 simple moving average filter attenuated high-frequency components, removing 97.4% of power in the 2–15 Hz noise band in the supplementary analysis. The estimated quiescent noise floor remained well below the deployment peak-detection threshold θ = 0.20 (Figure S2). These results support the use of the smoothed acceleration-derived signal for event-level peak detection in the present dataset, although they do not replace full error-propagation validation against external motion-capture ground truth.

### 3.6. Comparison with the Motion Energy Analysis Convention

To position the deployed pipeline within the established synchrony literature, we applied the windowed cross-correlation convention of Motion Energy Analysis [41,42] to the same 25 dyads using the parameters those authors recommend (1 min non-overlapping windows, ±5 s maximum lag, absolute-value aggregation, 100 window-wise pseudosynchrony surrogates).

**Observed vs. pseudosynchrony.** At the corpus level, observed MEA-style synchrony averaged 0.0736 ± 0.0093, and pseudosynchrony averaged 0.0654 ± 0.0048 (Figure 5e). The paired comparison was significant (*t*(24) = 3.76, *p* < 0.001; mean within-pair difference = +0.008; Cohen’s *d_z_* = 0.75). Separately, the dyad-specific observed-versus-surrogate standardized effect sizes averaged Cohen’s *d* = +1.07 ± 1.11, with 21 of 25 dyads exceeding *d* = 0 and 17 of 25 exceeding *d* = 0.5.

**Lack of cross-method rank-order convergence.** Although both methods showed above-chance synchrony at the group level, the per-dyad rank ordering produced by the two methods was essentially uncorrelated: the Pearson correlation between PDA Frequency and MEA |r| across the 25 dyads was r = +0.021 (*p* = 0.919; Figure 5f). Thus, the two methods agreed that synchrony exceeded chance at the group level but did not produce the same dyad ranking. This supports the view that distinct algorithmic families capture distinct facets of dyadic coordination [6,43].

To further test whether the two approaches could be treated as interchangeable dyad-level measures, PDA Frequency and MEA |r| were z-standardized across dyads and analyzed for agreement. The intraclass correlation for absolute agreement was near zero, ICC(2,1) = −0.02. Bland–Altman analysis showed a mean difference near zero but wide 95% limits of agreement, approximately −2.86 to +2.86 SD units (Figure S3). These results indicate that PDA Frequency and MEA |r| should not be interpreted as interchangeable dyad-level indices, even though both exceeded their respective chance baselines at the group level.

### 3.7. Summary

The evaluation provides four deployment-relevant findings. First, the modified Kinect phase-difference pipeline detected repeated event-level synchrony in all 25 free-conversation dyads while preserving a near-zero Lead–lag value appropriate for role-free dialog. Second, observed phase-difference metrics exceeded within-dyad surrogate controls, indicating temporal coordination above chance-level peak matching. Third, MEA-style cross-correlation applied to the same acceleration-magnitude signals also distinguished observed dyads from surrogate controls, aligning the present Kinect-derived signals with established nonverbal synchrony methodology. Fourth, dyad-level rank ordering was not shared by phase-difference and cross-correlation metrics, indicating complementary rather than interchangeable measures.

## 4. Discussion

### 4.1. Principal Findings

This article presented Synchrony Vision, an RGB-D sensor-based human motion analysis system for real-time monitoring and event-level analysis of interpersonal motion synchrony from markerless skeletal tracking. We described its sensing pipeline, signal-processing core, user interface, and deployment-condition evaluation. The contribution is twofold. On the system side, Synchrony Vision provides an operator-facing workflow that transforms RGB-D skeletal motion into acceleration-derived synchrony metrics while preserving exportable artifacts for external analysis. On the evidence side, the evaluation on 25 dyads of unconstrained Kinect-tracked free dialog (245,835 frames and 200.6 min wall-clock; 2681 events; phase differences computed from Time.csv timestamps) showed that the four user-facing metrics behaved coherently in the deployment regime: the aggregated phase-difference distribution was unimodal and centered near zero, event density exceeded circular-surrogate baselines, and dyad ordering was preserved across reasonable parameter neighborhoods. These findings extend prior controlled-task and accelerometer-comparison validation [32] by showing that the system produces stable and interpretable outputs in the type of role-free, naturalistic interaction for which it is intended.

### 4.2. Interpretability, Robustness, and Ecological Validity of the User-Facing Metrics

The phase-difference analysis detected 2681 synchrony events over 200.6 min of free conversation. This finding is important because the evaluation was not based on repeated, externally paced, or experimentally simplified rhythmic movements. Instead, the data were obtained from spontaneous, role-free dyadic conversations in which participants freely produced irregular and heterogeneous movement events, such as nods, posture shifts, listener responses, and speaking-related body movements. Nevertheless, Synchrony Vision produced interpretable Frequency, Lead–lag, Width, and Strength values across all dyads, consistent with prior phase-difference synchrony features used in communication and clinical studies [39,40]. These results indicate that the system can extend phase-difference analysis to markerless, timestamp-aware, free-dialog measurement.

The four metrics provide complementary information for interpreting the Result workspace. Frequency indicates how often synchrony events occur and serves as a reliable descriptor of event density across dyads, showing clear dyad-level variability and consistent behavior under parameter changes. Lead–lag summarizes the average signed temporal offset between paired movement events. Width captures the temporal dispersion of event timing, and Strength reflects the peakedness of the phase-difference distribution. Together, these metrics provide users with a multidimensional profile of interpersonal coordination rather than a single opaque synchrony score.

The parameter-robustness analyses further support field deployment. Although the absolute magnitude of Frequency changed predictably with the peak-detection threshold θ, smoothing window W, and pairing window $\tau_{max}$, the rank order of dyads was preserved across reasonable parameter neighborhoods. This invariance is important because it means that the substantive conclusion that one dyad shows more frequent synchrony than another does not depend on a single arbitrary parameter setting. It also suggests that rating thresholds defined in the INI file can be calibrated once for a deployment context, such as a clinic or classroom, and reused across sessions rather than requiring per-session tuning by non-expert operators.

The present evaluation therefore extends the earlier system paper by focusing on the design and implementation of the user-facing interface layer and by evaluating whether the user-facing synchrony metrics behave coherently under naturalistic free-conversation conditions [32]. The prior publication documented the implemented computation core and its feasibility. In contrast, the present study addresses a dyad-level deployment question: whether the four user-facing summary metrics behave coherently in their distribution, relation to chance, parameter dependence, and comparison to alternative synchrony formulations when applied to long, unconstrained conversations. Together, the findings indicate that Synchrony Vision can bridge algorithmic implementation and practical deployment by preserving the interpretability of phase-difference metrics under naturalistic free-conversation conditions.

### 4.3. Relationship with MEA-Style Cross-Correlation

The Motion Energy Analysis (MEA) framework developed by Ramseyer and Tschacher [41,42] is one of the most widely used approaches for quantifying nonverbal synchrony from video, particularly in psychotherapy research. Its analytical core is windowed cross-correlation of movement time series, typically using non-overlapping 1 min windows, a maximum lag of ±5 s, absolute-value aggregation, and pseudosynchrony controls [41,42,43]. Across independent samples, MEA studies have consistently shown that observed interactions exhibit stronger synchrony than pseudo-interactions, with standardized within-pair effect sizes in the approximate range of d = 0.20–1.02 [41,42].

The present Kinect-based analysis reproduced this core MEA pattern using acceleration-derived movement signals. Applying the same general windowed cross-correlation logic to the z-scored, smoothed acceleration magnitudes yielded above-chance synchrony. The paired group-level comparison produced Cohen’s *dz* = 0.75, whereas the mean dyad-specific observed-versus-surrogate standardized effect size was *d* = 1.07. These effect sizes are similar in magnitude to those reported in canonical MEA studies, suggesting that the exported Kinect-derived acceleration signals can serve as a useful substrate for MEA-style analyses. However, the raw synchrony coefficients should not be directly equated across modalities because MEA uses frame-differenced pixel-change motion energy, whereas the present system uses three-dimensional acceleration magnitude derived from skeletal tracking. Thus, convergence should be interpreted at the level of standardized observed-versus-surrogate effect size, not at the level of absolute correlation magnitude.

At the same time, the phase-difference and MEA-style analyses did not produce the same dyad-level ranking. PDA Frequency was essentially uncorrelated with MEA |r| (r = +0.021, *p* = 0.919), indicating that the two methods agree that synchrony exceeds chance at the group level but disagree about which dyads are most synchronized. This pattern is consistent with prior evidence that different synchrony algorithms may show limited convergent validity and may capture distinct facets of dyadic coordination [6,43]. The phase-difference pipeline identifies discrete motion events and quantifies their millisecond-scale temporal alignment within a ±1.0 s pairing window. In contrast, MEA-style cross-correlation integrates movement covariation across longer 60 s windows and is more sensitive to sustained changes in movement energy. Therefore, the two approaches should be interpreted as complementary measurement surfaces rather than interchangeable estimates of a single synchrony construct. We do not claim that the two methods measure the same quantity. They concur at the group level in that both methods exceed their respective chance baselines (PDA Cohen’s *dz* = 1.04 and MEA Cohen’s *dz* = 0.75), but they diverge at the per-dyad level: the intraclass correlation for absolute agreement on the standardized values is ICC(2,1) = −0.02, and a Bland–Altman analysis yields a mean difference near zero with 95% limits of agreement spanning roughly ±2.9 SD (Figure S3). Users should therefore treat PDA Frequency and MEA |r| as complementary indices and report which method and parameters were used.

This dissociation is consistent with a substantial body of work indicating that phase-based and amplitude-based measures index different facets of synchrony. In dynamical-systems terms, the Kuramoto framework reduces interacting oscillators to their phases, and its order parameter quantifies phase coherence specifically; because phase dynamics can be formally decoupled from amplitude dynamics, the timing of cyclic events and the co-variation in their magnitude are distinct descriptors of the same signals [44,45]. In neurophysiology, the same contrast is formalized as two “intrinsic coupling modes”—phase coupling of band-limited oscillations versus coupled fluctuations of signal envelopes—which differ in their dynamics, origins, and putative functions [46], and cortical phase- and amplitude-coupling patterns have been shown empirically to be non-redundant and to reflect at least partly distinct mechanisms [47]. The phase-difference algorithm, which times discrete movement events, is a phase-type measure, whereas Motion Energy Analysis, which correlates windowed motion-energy envelopes, is an amplitude-type measure. Low dyad-level convergence between these measures is therefore not necessarily anomalous; it may reflect their sensitivity to different representational levels of synchrony.

The same distinction is documented within the interpersonal-synchrony literature, where synchrony is commonly decomposed into its degree or intensity—captured by cross-correlation or coherence of movement amplitude—and its pattern or timing—captured by relative phase [48,49]. MEA-style windowed cross-correlation typically uses the absolute coefficient, reflecting the degree of co-movement while discarding the in-phase/anti-phase distinction [41]. Systematic comparisons report that different time-series measures of nonverbal synchrony lack convergent validity and instead index separable facets, to the point of yielding opposite associations with clinical variables depending on the measure used [50]. Accordingly, we interpret the near-zero PDA–MEA correlation as the expected signature of two complementary measurement families and report both rather than treating either as a gold standard [6].

This distinction has practical implications for system use. Researchers interested in the timing of discrete co-motion events, such as nods, gestural adjustments, postural shifts, or brief backchannel responses, should primarily use the phase-difference metrics exposed in the Result workspace. Researchers interested in slower, sustained dyadic coupling may instead use the exported acceleration files for MEA-style or other continuous-signal analyses. By exposing both interpretable phase-difference outputs and raw acceleration artifacts, Synchrony Vision supports multiple analytical lenses while clarifying what each method is expected to reveal.

### 4.4. Practical Implications

We stress that the present study did not measure clinical, behavioral, educational, rehabilitation, or HRI outcomes. Therefore, the following scenarios should be understood as operational examples of how the system could be used in future validation studies, not as validated effects of the proposed metrics. In clinical contexts, Synchrony Vision may provide a scalable tool for quantifying nonverbal social timing under naturalistic interaction conditions. Recent work using the phase-difference framework in autism spectrum disorder (ASD) showed that synchrony-related features can distinguish TD–TD and TD–ASD dyads, with TD–ASD dyads characterized by lower synchrony frequency, greater temporal variability, and weaker coherence. These findings suggest that phase-based metrics may provide candidate behavioral descriptors of atypical social coordination [40]. The present system extends this line of work by implementing a markerless, operator-facing platform that can export the same family of interpretable temporal synchrony metrics without requiring head-mounted or body-worn sensors. For example, a clinician could record the same child–partner dyad across successive intervention sessions under a fixed configuration, including the same joint, threshold, and frame-rate settings, and examine whether the per-minute rate or temporal consistency of aligned head-movement events changes over time. However, whether such changes correspond to clinical improvement or social-communication outcomes must be established separately using domain-specific measures.

In educational and HRI contexts, the system may also support future studies of interaction timing. For example, a teacher could compare the same dyad across two structured activities, such as teacher-led explanation and collaborative discussion, to examine whether the temporal alignment of head-movement events differs across interaction formats. In HRI studies, event-level phase-difference metrics could be used to evaluate whether different robot speech-timing, gesture-timing, gaze, or turn-taking strategies are associated with different patterns of human movement timing. Because Synchrony Vision estimates synchrony from markerless skeletal data, such studies could be conducted without requiring participants to wear sensors, which may be useful in educational robots, care robots, or rehabilitation robots where the interaction itself should remain natural and low burden. Nevertheless, these use cases require separate validation against learning gains, interaction-quality ratings, engagement measures, rehabilitation indices, or other domain-specific outcomes before the metrics can be used for decision-making.

Finally, the system’s exportable artifacts support reproducibility and methodological flexibility. Researchers can use the Result workspace for immediate inspection while also reanalyzing the exported participant-level acceleration data, frame-level timestamps, and synchrony outputs with independent pipelines. This is important because synchrony is a multidimensional construct rather than a single latent value. The present comparison with MEA-style analysis shows that different algorithms can agree at the observed-versus-surrogate level while producing different dyad-level rankings. Therefore, a useful deployment system should not force a single interpretation of synchrony, but should provide transparent outputs that support multiple analytical lenses. Synchrony Vision contributes to this goal by combining live monitoring, interpretable event-level temporal metrics, and compatibility with established synchrony analysis frameworks.

Generalizing the metrics to clinical, educational, rehabilitation, or HRI decision-making requires a separate validation step in which the metrics are related to domain-specific outcomes, such as standardized clinical scores, learning gains, rehabilitation progress measures, HRI engagement indices, or interaction-quality ratings collected in the target population. The present work provides the deployment-condition characterization that is a prerequisite for such validation, but it does not itself establish criterion validity for these outcomes.

### 4.5. Limitations

Several limitations should be noted. First and most importantly, this study does not establish criterion validity for the proposed metrics. The corpus contains no independent behavioral, psychological, educational, clinical, rehabilitation, or HRI outcome variables; therefore, Frequency, Lead–lag, Width, and Strength should be interpreted as signal-level descriptors of event-level temporal alignment, not as validated indicators of psychological constructs, interaction quality, clinical improvement, learning outcomes, or intervention effects. The present results establish only that movement events co-occurred in time more often than expected under a within-dyad chance baseline; whether these metrics relate to rapport, empathy, cooperation, social engagement, or other domain-specific outcomes remains a hypothesis to be tested with appropriate outcome measures.

Second, the metrics are configuration-relative and are comparable only under fixed joint and parameter settings. In the present evaluation, the metrics were computed from the head-joint signal, and cross-joint effects were not characterized. Similarly, the default ± 1.0 s pairing window should be interpreted as a literature-based and empirically reasonable deployment default for the present free-dialog corpus, not as a universally optimal timescale. Although the sensitivity analysis showed that Frequency increased mainly between 0.5 and 1.0 s and flattened beyond 1.0 s in the present dataset, synchrony in other populations or tasks may occur over longer or more variable timescales.

Third, although Time.csv-based analyses indicated that long timestamp gaps were rare and that maximum gaps remained below the pairing window, the deployed software logged per-frame timestamps but not SDK per-joint TrackingState values. Therefore, joint-confidence and occlusion flags could not be reconstructed for this corpus. Fourth, the smoothing and noise-floor analyses support the present peak-detection pipeline, but they do not constitute a full physical error-propagation analysis from Kinect position uncertainty to acceleration uncertainty. Fifth, no formal usability study was conducted with clinical, educational, or HRI practitioners; therefore, the practical usability of the interface for these target users has not yet been validated. Finally, the sample of 25 dyads, while comparable to many motion-synchrony studies, is moderate, and no cross-dataset or test–retest validation was performed.

### 4.6. Future Work

Several directions follow naturally from these limitations and results. First, formal usability studies should be conducted with target user populations, including clinicians, teachers, and HRI researchers. These studies should examine whether the metric cards and the millisecond-scale histogram support the inferences that users want to draw, using field-based task scenarios, task-completion measures, error rates, workload measures, and standardized usability instruments.

Second, the dyad-level corpus reported here should be extended to additional populations and tasks, including unidirectional speaker–listener configurations and clinical groups, to characterize how the distributions of Frequency, Lead–lag, Width, and Strength shift across contexts; preliminary results on autism spectrum disorder dyads using the same algorithmic family are already encouraging in this respect [40]. Future studies should also validate whether the default ± 1.0 s pairing window remains appropriate across different populations and interaction tasks. In groups with different motor or communicative characteristics, such as older adults, individuals with ASD, or patients with Parkinson’s disease, relevant synchrony may occur over longer or more variable timescales. Therefore, future deployments should validate the pairing window and other analysis parameters for each target population and task, and population- or context-specific parameter adjustment may be necessary.

Third, future deployments should improve tracking-quality logging and validation. In particular, the system should record SDK per-joint TrackingState values and selected-joint confidence for each frame, and Kinect-derived acceleration features should be compared with external motion-capture or inertial reference measurements to quantify position-to-acceleration error propagation.

Fourth, future studies should evaluate cross-dataset, test–retest, and cross-sensor generalizability. Although the present implementation used Kinect v2, the architecture is not tied to a specific sensor model; future implementations can replace the sensing backend with currently available RGB-D cameras, such as Azure Kinect or Orbbec Femto Bolt, or with deep-learning-based pose-estimation backends, provided that temporally aligned skeletal-position or body-pose signals can be exported for acceleration and phase-difference analysis.

Finally, future implementations should extend the sensing architecture beyond RGB-D skeletal tracking by integrating multimodal streams, such as audio-visual nonverbal behavior, multisensory interaction cues, and physiological signals. Such multimodal fusion, together with data-driven and AI-based methods, may improve robustness and adaptability in future versions, for example through denoising, adaptive thresholding, learned event detection, joint selection, or multimodal feature extraction [51,52,53,54,55].

## 5. Conclusions

Synchrony Vision provides a markerless, operator-facing platform for RGB-D sensor-based human motion analysis of interpersonal synchrony. By combining real-time monitoring, phase-difference analysis, and a two-workspace interface, the system is designed to support a workflow from measurement to interpretation without requiring a separate offline processing pipeline. Its live tracking feedback, real-time acceleration visualization, millisecond-scale phase-difference histogram, four interpretable synchrony metrics, and exportable acceleration, timestamp, and synchrony artifacts support both immediate inspection and reproducible downstream analysis. The evaluation on Kinect-tracked free-dialog dyads showed that the system can generate interpretable event-level temporal synchrony outputs under naturalistic conversation conditions. Overall, Synchrony Vision illustrates how RGB-D skeletal sensing can be extended from individual motion capture to event-level temporal analysis of interpersonal motion coordination, providing a transparent and extensible measurement platform with potential for future applications in education, clinical, rehabilitation, and human–robot interaction research, contingent on domain-specific validation against outcome measures.

## Data Availability

The participant acceleration data obtained through the evaluation sessions, together with the associated Time.csv timing files, have been deposited in a public repository on the Open Science Framework (OSF) and are openly available at https://osf.io/eb67k/ (accessed on 9 July 2026).

## Supplementary Materials

The following supporting information can be downloaded at: https://www.mdpi.com/article/10.3390/s26144445/s1, Figure S1: Frame-time and tracking continuity; Figure S2: Noise conditioning and error propagation; Figure S3: Cross-method agreement between PDA and MEA.
