# Supplementary material for: Synchrony Vision: An RGB-D Sensor-Based System for Real-Time Monitoring and Event-Level Analysis of Interpersonal Motion Synchrony
Source: Sensors (Basel). 2026 Jul 13;26(14):4445. doi: 10.3390/s26144445 (PMC13417634; doi:10.3390/s26144445)
Supplement: Supplementary file 1 [file sensors-26-04445-s001.zip › sensors-4393115-supplementary.pdf]

S1. Frame-Time and Export-Quality Analysis

To assess whether timestamp irregularities could affect event-level synchrony estimation, we analyzed Time.csv for all 25 dyads. For each dyad, we computed inter-frame intervals, median and mean effective frame rates, and the maximum timestamp gap. We also verified row alignment between Time.csv and the participant-level acceleration files. Figure S1 summarizes the frame-time characteristics of the exported recordings.

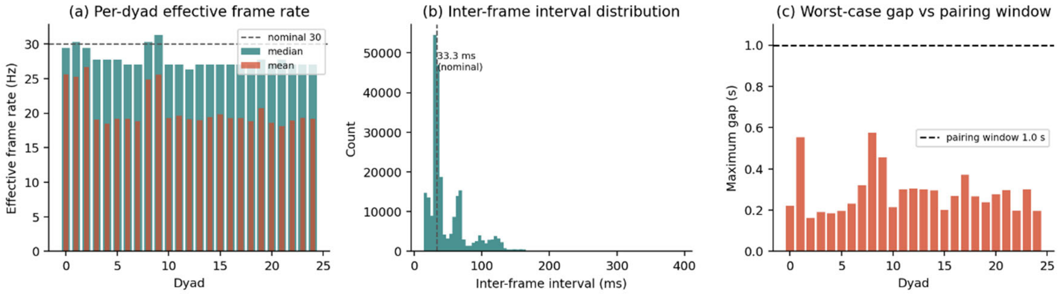

Figure S1. Frame-time characteristics of the exported recordings.

(a) Per-dyad median and mean effective frame rates computed from Time.csv. The dashed line indicates the nominal Kinect frame rate of 30 Hz.

(b) Distribution of inter-frame intervals across all dyads. The dashed vertical line indicates the nominal 33.3 ms interval.

(c) Maximum timestamp gap for each dyad relative to the ±1.0 s phase-difference pairing window. The maximum gap in every dyad remained below the pairing window.

S2. Smoothing and Noise-Floor Analysis

Because the acceleration-derived signals were computed from Kinect skeletal positions through finite differences, we examined the effect of the W = 11 simple moving average filter and the separation between quiescent noise and the peak-detection threshold. Figure S2 shows the filter frequency response, the reduction in high-frequency power, and the distribution of quiescent noise-floor estimates relative to $\theta = 0.20$.

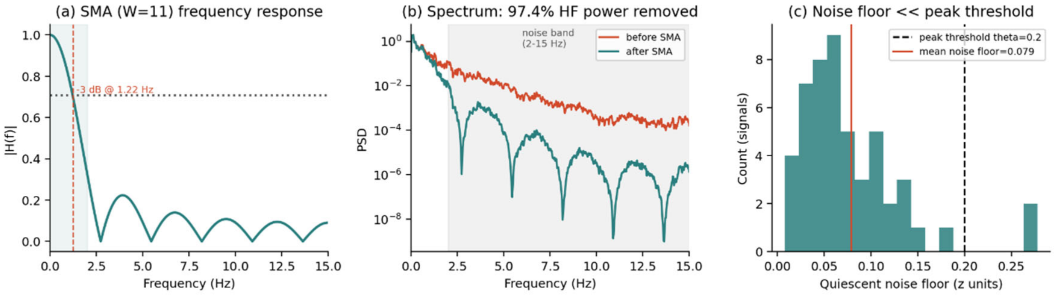

Figure S2. Smoothing and noise-floor analysis of Kinect-derived acceleration signals.

(a) Frequency response of the W = 11 simple moving average filter. The dashed line indicates the −3 dB cutoff.

(b) Power spectral density before and after smoothing, showing attenuation of high-frequency components in the 2–15 Hz noise band.

(c) Distribution of quiescent noise-floor estimates relative to the deployment peak-detection threshold $\theta = 0.20$. The estimated noise floor remained below the threshold.

S3. Cross-Method Agreement between PDA and MEA

To evaluate whether PDA Frequency and MEA |r| could be treated as interchangeable dyad-level synchrony indices, both measures were z-standardized across dyads and compared using Pearson correlation, ICC (2,1) for absolute agreement, and Bland–Altman analysis. Figure S3 summarizes the cross-method agreement analysis.

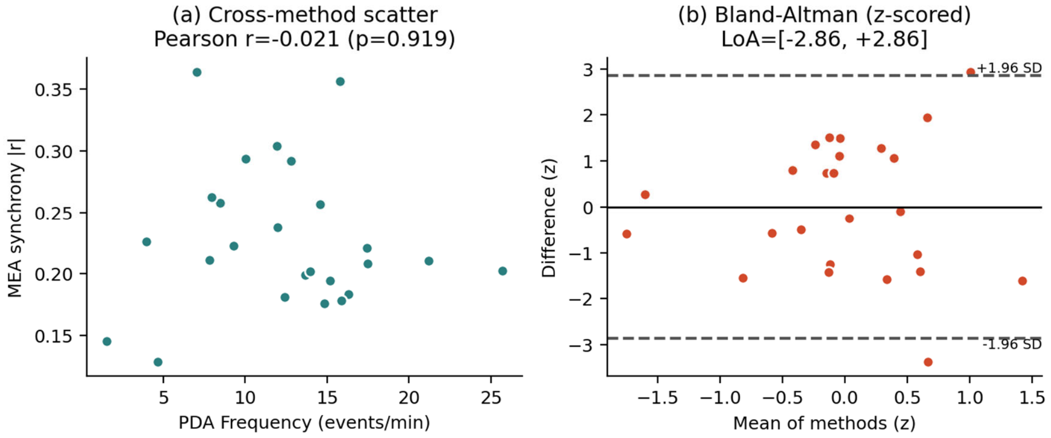

Figure S3. Cross-method agreement between phase-difference analysis and MEA-style synchrony.

(a) Scatterplot of PDA Frequency and MEA |r| across the 25 dyads, showing minimal dyad-level convergence.

(b) Bland–Altman plot of z-standardized PDA Frequency and MEA |r| values. The wide limits of agreement indicate that the two methods should not be treated as interchangeable dyad-level indices.
